# Evaluation of the complexity of indoor air in hospital wards based on PM2.5, real-time PCR, adenosine triphosphate bioluminescence assay, microbial culture and mass spectrometry

**DOI:** 10.1186/s12879-019-4249-z

**Published:** 2019-07-19

**Authors:** Shao Ling, Liu Hui

**Affiliations:** College of Medical Laboratory, Dalian Medical University, No.9 West Section Lvshun South Road, Dalian, 116044 China

**Keywords:** PM2.5, Real-time PCR, ATP bioluminescence assay, Passive sedimentation, Microbial ecology, MALDI-TOF MS

## Abstract

**Background:**

The aim of this study was to establish a set of assessment methods suitable for evaluating the complex indoor environment of hospital wards and to ascertain the composition of bacteria and microbial ecology of hospital wards.

**Methods:**

Colony-forming units (CFUs), PM2.5 detection, real-time PCR, and adenosine triphosphate (ATP) bioluminescence assay were employed to evaluate the complexity of indoor air in 18 wards of nine departments in a hospital and two student dormitories in a university. Subsequently, the microbial samples were quantified and identified using matrix-assisted laser desorption/ionization time-of-flight mass spectrometry (MALDI-TOF MS).

**Results:**

Although the studied indices were relatively independent, the PM2.5 content was correlated with bacterial CFUs determined by passive sedimentation method, bacterial and fungal counts measured by real-time PCR, and ATP bioluminescence assay. The composition of microorganisms in the air of hospital wards differed from that in the air of student dormitories. The dominant genera in hospital wards were *Staphylococcus* (39.4%), *Micrococcus* (21.9%), *Corynebacterium* (11.7%), *Kocuria* (4.4%), *Bacillus* (2.9%), *Streptococcus* (1.6%), *Moraxella* (1.6%), and *Enterococcus* (1.3%), and the microbial ecology differed between Respiration Dept. III and other hospital departments. Additionally, 11.1 and 27.3% of bacteria in hospital wards and student dormitories were not identified, respectively.

**Conclusions:**

Assessment of environmental quality of hospital wards should be based on comprehensive analysis with multiple indicators. There may be imbalances in the microbial diversity in the hospital wards, therefore, monitoring of the environmental quality of hospitals is important in the prevention of nosocomial infections.

**Electronic supplementary material:**

The online version of this article (10.1186/s12879-019-4249-z) contains supplementary material, which is available to authorized users.

## Background

The indoor environment of hospitals, with higher risk of microbial contamination, is complicated and different from other occupational or residential indoor settings. Most of the hospitalized patients spend two-third of their time in wards every day; in particular, bed-ridden patients spend almost all their time in the ward. Therefore, the air quality of hospitals may be an important risk factor affecting the health of patients, and assessment of hospital environment is particularly significant.

Airborne microflora in hospital as a potential cause of hospital infections has been the subject of numerous studies. Some researches have been showed that environmental contamination may promote the infection and transmission of healthcare-associated pathogens, including methicillin-resistant *Staphylococcus aureus* (MRSA), vancomycin-resistant *Enterococcus spp.* (VRE), *Clostridium difficile*, *Acinetobacter* spp., and norovirus [1, 2]. Also, some studies suggested that if the prior room was occupied by several important healthcare-associated pathogens, the risk of acquiring these bacteria by a new admission was significantly increased [3–6]. In addition, bioaerosol infection is common in hospitals and recognized to be the causation of agents and illness such as aspergillosis and tuberculosis, as a result, the levels, sources and characteristics of bioaerosols have been extensively investigated [7–11]. PM2.5 is an air pollutant with a diameter of ≤2.5 μm, which can be easily inhaled and can penetrate deep into the airway [12]. Many studies have confirmed that inhalation exposure to ambient PM in the environment, especially PM2.5, is related to certain systemic diseases and cancer [13–15]. Long- and short-term exposure to PM2.5 has been consistently associated with a number of outcomes, including mortality, cardiovascular and cerebrovascular events and lung cancer [16–20]. Calderón-Garcidueñas et al. highlighted that air pollution can be considered as a risk factor for both Alzheimer’s disease and Parkinson’s disease [21]. Currently, although the pathogenicity of PM2.5 in aerosol chemistry and physics has been extensively studied, a little is known about the inhalable biological particles such as bacteria, fungi, viruses, pollen, and cell debris [22–24]. In addition, airborne PM2.5 in indoor environments has been widely investigated because of its ability to cause adverse health effects [25]. A study conducted in the USA about hospital nurses confirmed that PM2.5 was related to the risk of all-cause mortality [26]. Hence, monitoring of indoor environmental quality is the first step to reduce the chance of nosocomial infections. The traditionally applied methods, to assess microbial air contamination and cleanliness on surfaces in hospital are based on microbiological cultural technology, including active, passive and surfaces sampling [27]. In recent decade, adenosine triphosphate (ATP) bioluminescence assay, which was used to assess environmental surface cleanliness has been proposed [28]. Previous studies have shown that the number of microorganisms that can be cultured only accounts for < 5% of the total microorganisms in air [29–31]. Therefore, relying solely on the method of cultivation may not help in accurate assessment of hospital environment. Hence, comprehensive indicators, including fine particles, total microorganisms (including dead microorganisms), culturable microorganisms, and number of living cells, are needed to analyze the complex hospital wards environment.

Conventional microbiological methods for bacterial identification involve culture, microscopic examination, and biochemical testing. Although these procedures can be accurate and reliable, they are time-consuming and require well-trained technicians to interpret the results [32]. Currently, whole genome sequencing (WGS), an analytical technology that allows sequencing of the whole genomic content of bacteria, would be the best approach to bacterial identification. Matrix-assisted laser desorption/ionization time-of-flight mass spectrometry (MALDI-TOF MS) has emerged as a tool for microbial identification and diagnosis owing to its specificity, high speed, and low costs. This technology has been widely used for microbial identification and strain typing, detection of antibiotic resistance, detection of blood and urinary tract pathogens, and detection of water- and food-borne pathogens, etc. [33].

In the present study, we established a set of assessment methods suitable for evaluating the complex indoor environment of hospital wards by using both cultivation and non-cultivation approaches. Subsequently, MALDI-TOF MS was employed to identify the isolated organisms to assess the microbial ecology of the studied environment.

## Methods

### Setting and sampling

The hospital selected in this study is a comprehensive teaching hospital with three buildings and 1,788 beds in Northeast China. The wards of two hospital buildings (Nos. 1 and 9 buildings), which have the same central HVAC system and the area of each ward was 30 m^2^, were selected for the investigation. The two buildings were completed in 2016 and 2000, respectively. To compare the differences between the hospital and natural environment, two student dormitories in a university were selected as control. The indoor environmental indicators were monitored in the general hospital in spring between 1/3/2018 and 15/3/2018. The research was approved by the Ethical Committee of Dalian Medical University, China.

### Detection of PM2.5 and PM10

The indoor PM2.5 concentrations were detected using optical particle counter PC-5A (Laser Research Institute, Jiangsu, China), which employs light scattering technology to deliver real-time measurements [34]. In addition to the seventh floor (Obstetrics Dept.) in No. 1 building, five wards of each department from floor 6 to 17 were selected for PM10 detection. Likewise, in No. 9 building, five wards of each department from floor 1 to 11 were selected for PM10 detection. In No. 1 building, three departments with the highest concentration of PM10, namely, General Surgery Dept. VI Ward, GI Surgery Dept. Ward, and GI Dept. I Ward, and two departments with the lowest concentration of PM10, namely, ENT Dept. Ward and Nephrology Dept. Ward, were selected. In No. 9 building, two departments with the highest concentration of PM10, namely, Respiration Dept. I Ward on floor 11 and Respiration Dept. III Ward on floor 9, and two departments with the lowest PM10 concentration, namely, Neurology Dept. VI Ward on floor 3 and Neurology Dept. VII Ward on floor 2, were chosen. Among these nine selected departments, two wards were chosen for each department (totaling to 18 wards), and their PM2.5 content was ascertained. Two measured factors, namely, the use of a humidifier and presence of houseplants, were invariant and therefore not included in the analysis. The air temperature and relative humidity were measured for the duration of the sampling period. The student dormitories, as control, were simultaneously subjected to PM2.5 and PM10 analysis.

### Passive sedimentation and dust sample collection

In the 18 wards of the selected nine departments, the bacterial and fungal cultures were accomplished by traditional passive sedimentation method according to the Hospital Sanitary Disinfection Standard (GB15982–2012). The inner, middle, and outer diagonal lines were set up at three points. The inner and outer points were located at 1 m from the wall and at a height of 0.8 m. Three Columbia blood agar plates (BIOMERIEUX, France) for bacteria and three Sabouraud medium plates (Yancheng biology, China) for fungi were placed at the sampling points in each ward. During sampling, the plates were opened for 5 min.

A modified method based on the standard ASTM (method E1728–03) [35], combined with the protocol developed by Yamamoto [36] and Rachel I. Adams [37], was employed to collect indoor dust. Sterile cotton swabs pre-wetted with 0.15 M NaCl and 0.05% Tween 20 sampling buffer were swabbed for 5–10 s on the four corners of the floor, each with an area of 5 × 5 cm, to collect the dust. Then, the swabs were placed in sampling tubes and stored at − 20 °C until DNA extraction. In general, four swabs were obtained for each ward. During the DNA extraction process, two swabs from the same ward were combined into one DNA extraction reaction mixture to obtain sufficient biomass for analysis.

### DNA extraction

The head of the swab was cut using sterile scissor (sterilized using 75% alcohol) and placed into a 2-ml DNeasy PowerSoil extraction tube (Hilden, Germany). Then, solution C1 was added to the extraction tube and incubated at 65 °C for 10 min. After incubation, the tube was vortexed with a vortex mixer for 20 min, and the DNA was extracted using DNeasy PowerSoil Kit (Hilden, Germany) according to the manufacturer’s protocol.

The bacterial universal primer p1370/p201 [38] and fungal universal primer NL1/260R [39] were used to amplify the fragment of bacterial 16S rDNA subunit and fungal 28S rDNA subunit, respectively. All the primers used were synthesized by Takara (Japan), and their sequence details are listed in Table 1.

**Table 1:** Sequences of the primers used

Name of the targeted species	Sequences	
Bacteria	Forward primer p1370	5′-AGICCCGIGAACGTATTCAC-3′
	Reverse primer p201	5′-GAGGAAGGIGIGGAIGACGT-3′
Fungi	Forward primer NL1	5′-GCATATCAATAAGCGGAGGAAAAG-3′
	Reverse primer 260R	5′-TTAGCTTTAGATGRARTTTACCACC-3′

The amplified fragments were run on a Real-Time PCR System (Thermal Cycler Dice Real Time System TP800, Takara, Japan) containing 12.5 μl of SYBR Premix Ex Taq II (Tli RNaseH Plus) (2×) (Takara, Japan), 0.5 μl of each 0.4 μM primer, 2 μl of genomic DNA, and water to a final volume of 25 μl. For bacteria, the thermal cycling conditions consisted of an initial denaturation at 95 °C for 30 s, followed by 40 cycles of denaturation at 95 °C for 5 s, annealing at 55 °C for 15 s, and extension at 72 °C for 10 s. For fungi, the PCR conditions were as follows: 95 °C for 30 s, followed by 40 cycles of denaturation at 95 °C for 10 s, annealing at 60 °C for 35 s, and extension at 72 °C for 20 s. Standard curves for bacteria and fungi were based on extraction of a known quantity of *Escherichia coli* and *Aspergillus oryza*e spores, respectively.

### ATP bioluminescence assay

For ATP bioluminescence assay, Clean-Trace TM NGI instrument, along with Clean-Trace TM Test Kit (3 M, UK), was used. In the 18 wards of the nine selected departments and two student dormitories, a luciferase-containing sampling swab was swabbed at an area of 10 × 10 cm at the end of each bed, and the swab was inserted into the Clean-Trace tube. Then, the handle was pushed so that the sampling rod was completely inserted into the bottom. After shaking for 5 s, the bioluminescence was measured immediately.

### Identification by MALDI-TOF MS

Single colonies freshly grown on agar plates were picked and smeared as a thin film directly onto a MALDI steel target plate. Subsequently, 10 μl of 70% formic acid were added and the microbial film was overlaid with 10 μl of MALDI HCCA matrix (50% acetonitrile, 2.5% trifluoroacetic acid, and 47.5% water), as recommended by the MALDI-TOF MS manufacturer. The sample-matrix mixture was dried at room temperature and analyzed using a MALDI-TOF MS analyzer (Bruker Daltonics, Bremen, Germany). The data were processed using MALDI Biotyper 2.3 software (Bruker Daltronics) and the spectra were compared with reference libraries for bacterial identification. A total of three rounds of MALDI-TOF MS were performed. The unidentified bacteria in the first round were subcultured for 24 h and a second round of MALDI-TOF MS identification was performed. The bacteria that still remained unidentified were subcultured for a further 24 h and a third round of MALDI-TOF MS was performed.

### Statistical analysis

The mean values of CFUs, PM2.5 and PM10 contents, CT values, and ATP contents in the 18 wards and two student dormitories were calculated, and correlation analyses were performed to determine the relationships among these index. Wilcoxon sum rank test was used to compare the biomass of bacteria and fungi in the wards, and one-sample *t*-test was employed to assess the difference between the means of wards and dormitories. The statistical analysis was performed using SPSS 24.0 statistical software, and *p* < 0.05 was considered significant.

## Results

### Contents of PM2.5 and PM10, bacteria, fungi, and ATP in the study sites

**Fig. 1:**
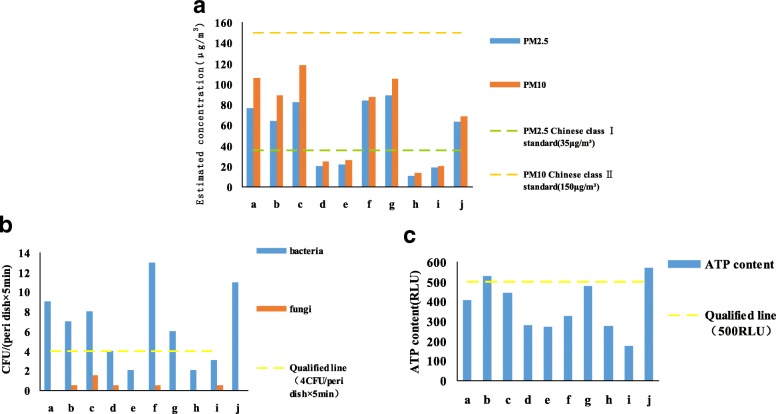
Comparison of the concentrations of PM2.5, PM10 (**a**), bacterial CFUs, fungal CFUs (**b**), and ATP content (**c**) in the examined wards and dormitories. a: General Surgery Dept.VI Ward; b: Gastrointestinal Surgery Ward; c: GI Dept. IWard; d: ENT Ward; e: Nephrology Ward; f: Respiration Dept.IWard; g: Respiration Dept.III Ward; h: Neurology Dept.VI Ward; i: Neurology Dept.VII Ward; j: Student dormitories

According to the Chinese Indoor Environment and Environmental Product Quality Supervision and Inspection Center, which issued the first indoor environment and development trend of the indoor environment industry, the indoor PM2.5 test standard is the first-class concentration limit of the “Ambient Air Quality Standard” published in 2012, and the daily average PM2.5 concentration is 35 μg/m^3^. In the present study, the concentration of PM2.5 in the wards of four departments, including ENT Dept., Nephrology Dept., Neurology Dept. VI, and Neurology Dept. VII, were in accordance with the standard. However, the concentrations of PM2.5 in the wards of other departments, including General Surgery Dept. VI, GI Surgery Dept., GI Dept., Respiration Dept. I, and Respiration Dept. III, as well as student dormitories exceeded the standard. Among them, the concentration of PM2.5 (89 μg/m^3^) in the Respiration Dept. III Ward was the highest, whereas the lowest PM2.5 concentration (10 μg/m^3^) was noted in the Neurology Dept. VI Ward. According to the “Ambient Air Quality Standard” (GB 3095–2012), the average daily PM10 concentration limit is 150 μg/m^3^. The PM10 concentration in all the wards and dormitories examined in this study did not exceed the limit, and the highest PM10 content was noted in the GI Surgery Dept. Ward (118 μg/m^3^), and the lowest content of PM10 was found in the Neurology Dept. VI Ward (13 μg/m^3^) (Fig. 1A).

In accordance with the Hospital Sanitary Standards for Sanitization (15982–2012), the average number of bacterial colonies in the Class III general wards was < 4 CFU/petri dish× 5 min. The number of bacterial colonies detected in the wards of four departments, including ENT Dept., Nephrology Dept., Neurology Dept. VI, and Neurology Dept. VII, was consistent with the standard, and the qualification rate was 44.4%; however, the bacterial colonies quantified in the wards of other departments examined exceeded the standard. Furthermore, as student dormitories do not qualify as medical environment, Indoor Air Quality Standard (GB/T18883–2002) was applied for the assessment, which stipulates < 2500 CFU/m^3^ as the standard total number of bacteria in indoor air. The total number of bacteria can be converted to the concentration of microorganisms in air, according to ORM’s formula as follows: C = 100/A × 5/t × 1000/10 × *N* = 50000 N/At, where C is the microbial concentration in air (CFU/m^3^), A is the culture dish area (cm^2^), t is the sampling time (min), and N is the number of colonies in each dish (CFU). In the present study, the concentration of microorganisms in the student dormitories was 1730 CFU/m^3^, which was consistent with the standard (Fig. 1B). In addition, the concentrations of microorganisms in the selected nine hospital departments were also calculated.

Based on the standard provided by the ATP bioluminescence manufacturer, the qualified, critical, and unqualified detection values for the ATP content were 0–250, 251–499, and ≥ 500 RLU, respectively, in the general ward area (patients’ active area, rehabilitation area, and family waiting room). Furthermore, Neurology Dept. VII was the only department that presented qualified values, whereas the General Surgery Dept. VI, GI Dept. I, ENT Dept., Nephrology Dept., Respiration Dept. I, Respiration Dept. III, and Neurology Dept. VI exhibited critical values. The unqualified values were noted in the GI Surgery Dept. and student dormitories (Fig. 1C).

### Estimation of bacterial and fungal biomass in the studied sites by real-time PCR

**Fig. 2:**
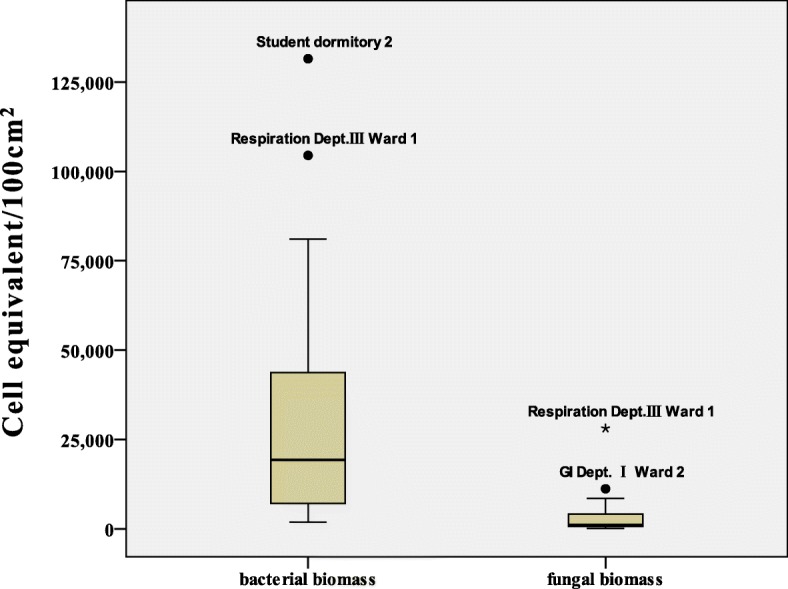
Estimation of bacterial and fungal biomass in the studied sites by real-time PCR

The bacterial and fungal biomass in the 18 wards and two student dormitories was estimated by real-time PCR. The fungal biomass estimates were in the range of 96–28,117 (median: 3799) cell equivalents/100 cm^2^, while bacterial biomass estimates, ranged from 1,858 to 131,514 (median: 32,488) cell equivalents/100 cm^2^, were generally greater than fungal biomass (*p* < 0.01). The biomass of bacteria in the Respiration Dept. III Ward 1 and student dormitory 2 was higher than that in the other wards and dormitories. The fungal biomass in the GI Dept. I Ward 2 was higher than that in the other wards, and was significantly higher in the Respiration Dept. III Ward 1 than that in the other wards and student dormitories (Fig. 2).

### Estimation of balance of microbial ecology of the studied sites using real-time PCR

We used the ratio of fungal biomass to concentration of microorganisms (F/M ratio) to judge whether there was an imbalance in the microbial ecology of the selected hospital departments. The results showed the F/M ratio for Respiration Dept. III was greater than other wards and dormitories (*p* = 0.031), indicating that there may have been disorders in the microbial ecology.

### Correlation among PM2.5 and PM10, CT values of bacteria and fungi, CFUs, and ATP content

It can be seen from Table 2 that the CT values for bacteria measured by real-time PCR were negatively correlated with the ATP content (*p* < 0.05); in other words, the bacterial concentration detected by real-time PCR was related to the number of living microorganisms. However, the bacterial and fungal counts determined by passive sedimentation method were not related to the ATP content. Furthermore, PM2.5 content was negatively correlated with the CT values for bacteria and fungi determined by real-time PCR (*p* < 0.05), and significantly correlated with the bacterial counts detected by passive sedimentation method (*p* < 0.01). In addition, the PM2.5 and ATP contents were positively correlated (*p* < 0.05). The content of PM10 was not related to the CT values for bacteria and fungi detected by real-time PCR, but was positively correlated with the bacterial counts measured by passive sedimentation method (*p* < 0.05), and had a significant positive correlation with the ATP content (*p* < 0.01). Thus, when compared with PM10, PM2.5 was a better indicator reflecting the microbial content in the air of the examined wards.

**Table 2:** Correlation among PM2.5 and PM10, CT values of bacteria and fungi, CFUs, and ATP content

	CT values of fungi	PM2.5	PM10	ATP content	CFUs of bacteria	CFUs of fungi
CT values of bacteria	0.587	−0.657^a^	−0.558	− 0.743^a^	−0.615	0.219
CT values of fungi	–	–	−0.608	–	–	−0.157
PM2.5	−0.666^a^	–	0.969^b^	0.759^a^	0.792^b^	0.100
PM10	–	–	–	0.777^b^	–	0.162
ATP content	−0.587	–	–	–	0.595	0.016
CFUs of bacteria	−0.388	–	0.692^a^	–	–	0.169

^a^Significant correlation at 0.05 level (bilateral);

^b^Significantly related at 0.01 level (bilateral)

### Bacterial characterization

The bacterial colonies isolated from the 18 hospital wards and two student dormitories were identified using MALDI-TOF MS. Among the 315 strains collected from hospital wards, 280 (88.9%) were identified, whereas 11.1% remained unidentified. Of the 66 strains collected in student dormitories, 48 (72.7%) were identified, whereas 27.3% remained unidentified. The specific results are shown in Table S3 (see Additional file 1).

### Analysis of dominant microbial genera in hospital wards

**Fig. 3:**
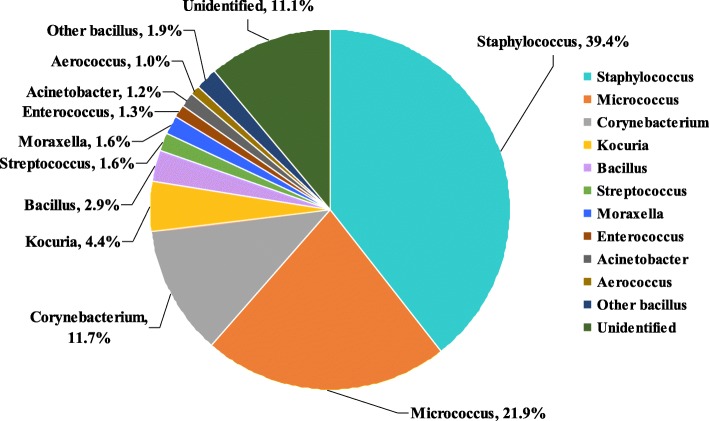
Distribution of dominant airborne microorganisms in the hospital environment

The dominant microbial genera comprised *Staphylococcus* (39.4%), *Micrococcus* (21.9%), *Corynebacterium* (11.7%), *Kocuria* (4.4%), *Bacillus* (2.9%), *Streptococcus* (1.6%), *Moraxella* (1.6%), and *Enterococcus* (1.3%). The dominant microorganisms in hospital wards are shown in Fig. 3.

### Comparison of dominant microbial genera in hospital wards and student dormitories

**Fig. 4:**
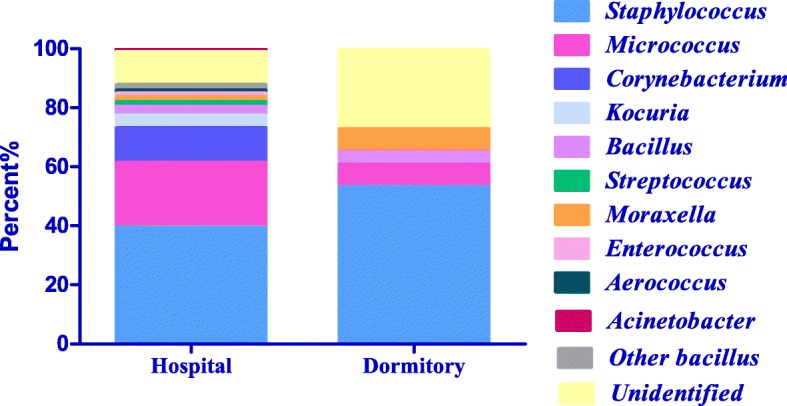
Dominant genera in hospital wards and student dormitories

The microbial composition of hospital wards differed from that of student dormitories. The dominant microbial genera in hospital wards were *Staphylococcus*, *Micrococcus*, *Corynebacterium*, *Kocuria*, *Bacillus*, *Streptococcus*, and *Enterococcus*, whereas those in student dormitories were *Staphylococcus*, *Micrococcus*, *Bacillus*, and *Moraxella* (Fig. 4). Additionally, a small number of bacilli, such as *Cellulosimicrobium cellulans, Pseudomonas oryzihabitans*, *Exiguobacterium aurantiacum*, *Curtobacterium flaccumfaciens*, and *Arthrobacter pascens*, and Gram-positive cocci, including *Aerococcus viridans*, were identified in the hospital wards. Unidentified bacteria accounted for 11.1 and 27.3% of the bacteria isolated from hospital wards and student dormitories, respectively.

## Discussion

Air quality in hospitals is likely to be a significant risk factor for the health of patients who visit hospitals. In this study, we employed four different indicators to assess the hospital environment by using both cultivation and non-cultivation approaches. Examination of the correlation among passive sedimentation method, real-time PCR, and ATP bioluminescence assay in wards and dormitories revealed that the bacterial and fungal CFUs measured by passive sedimentation method were not correlated with the ATP content, whereas the CT values for bacteria determined by real-time PCR were negatively correlated with the ATP content (*p* < 0.05). In other words, the bacterial content detected by real-time PCR was related to the number of living microorganisms in the wards. Furthermore, the CT values detected by real-time PCR were not correlated with CFUs of bacteria and fungi ascertained by passive sedimentation method, suggesting that the two methods were relatively independent, resulting from the various growth conditions of microorganisms and most of the microorganisms are not culturable, while the real-time PCR method can reflect the content of all microorganisms including cultivable and non-cultivable bacteria. Also, the efficiency of DNA extraction, the presence of PCR inhibitors and other factors have been important considerations for real-time PCR results, thus, microbial culture should be combined with real-time PCR for better evaluation of the environment complex of hospital general wards. In addition, as PM2.5 and PM10 detection, bacterial and fungal counts by real-time PCR, CFUs determination by passive sedimentation method, and ATP bioluminescence assay were not completely correlated with each other, it is necessary to combine various indicators to comprehensively analyze the complicated hospital wards environment.

In the present work, PM2.5 was found to have certain correlation with the bacterial and fungal content determined by real-time PCR, bacterial CFUs measured by passive sedimentation method, and ATP bioluminescence, suggesting that PM2.5 can indirectly reflect bacterial and fungal contamination in hospital wards. These results are consistent with the findings of Chen Cao et al. [40], who provided sequence-based evidence for the existence of inhalable microbial allergens and pathogen species in an open environment, and revealed that the relative abundance of these pollutants appeared to increase with increasing PM pollution. Bacteria and fungi are biological compositions of PM2.5 and most of the microorganisms in air adhere to particles of different sizes, and the pathogens in PM2.5 are considered to be the cause of various allergic and respiratory diseases. In the present study, the detection of PM2.5 content in the wards not only reflected PM pollution in the hospital environment, but also indirectly revealed the evaluation of microbial contamination in the ward. Therefore, PM2.5 detection is more important for assessing the environmental quality of hospital wards. In this study, we ascertained four comprehensive indicators using cultivation and non-cultivation approaches to assess the complex indoor environment. PM2.5 was also recommended for the evaluation of the hospital environmental quality because it is simple and has correlations with other indicators.

MALDI-TOF MS was used in this study to identify the bacterial composition in complex hospital wards and student dormitories. The dominant genera in the air of hospital wards differed from those in the student dormitories, and all of the identified *Staphylococcus* species were coagulase-negative staphylococci (CoNS). CoNS are typical opportunists and major nosocomial pathogens that have a substantial impact on human health. In particular, CoNS infections are associated with the use of implanted foreign bodies and central venous catheters, which are indispensable in modern medicine [41]. After their insertion, foreign bodies can get colonized by CoNS, resulting in severe burdens of medicine and economy [42]. In the present study, *S. epidermidis*, *S. hominis, S. haemolyticus*, and *S. capitis* were the major CoNS detected in hospital wards. In particular, the prevalence of *S. haemolyticus* was highest (22.7%) in the Respiration Dept. I. It must be noted that *S. haemolyticus*, which causes septicemia, peritonitis, otitis, and urinary tract infections, is the second most frequently isolated CoNS in human blood cultures [43–46]. Various studies have also reported high levels of antimicrobial resistance in *S. haemolyticus* [47, 48]; specifically, *S. haemolyticus* resistance to methicillin is on the rise, which has become a clinical challenge for clinicians. In the present study, the prevalence of CoNS in hospital wards differed from that in dormitories. The prevalence of *S. capitis* was the highest in Neurology Dept. VI Ward, accounting for nearly 31%, whereas *S. hominis* was dominant in dormitories. Colonization of different parts of the skin and mucosal membranes of the human body is a key source of endogenous CoNS infections. Therefore, to reduce the incidence of nosocomial infections and transmission of CoNS in hospital, measures including adequate sterilization of environmental surfaces and medical instruments, and sufficient hand hygiene should be adopted.

In this study, some rare genera, including *Kocuria* spp., were detected in the hospital*. Kocuria* spp. are commensals of humans, animals and the environment which can be found in drinking water, sediments, seeds, and fermented foods [49]. Although there are currently 18 known species of the genus *Kocuria*, only *K. kristinae*, *K. varians*, *K. marina*, *K. rhizophila,* and *K. rosea* have been recognized to cause opportunistic infections [50]. A recently identified *K. rosea* strain (BS1) has been reported to be capable of producing an exopolysaccharide (called Kocuran), which can inhibit the proliferation of phytohemagglutinin-stimulated human peripheral blood mononuclear cells and suppress complement-mediated hemolysis [51]. *Kocuria* spp. are normal flora of human skin and oral cavity, and are usually considered to be laboratory contaminants, in particular, their pathogenic potential is often ignored once isolated in clinical specimens. In addition, *Kocuria* spp. are generally misidentified as CoNS in the clinical microbiology laboratories owing to the lack of advanced techniques such as 16S rRNA and MALDI-TOF-MS [52]. In a recent study containing 12 pediatric patients suffering from debilitating conditions such as acute leukemia and premature birth, seven were confirmed to have *K. kristinae* bloodstream infection, thus highlighting the significance of *Kocuria* spp. in causing niosocomial infections [53]. However, another research reported a case of an immunocompetent girl, who suffered from endocarditis/sepsis by *K. rosea,* suggesting that immunocompromise is not an essential condition in all the reported cases [54]. Nevertheless, as diseases caused by *Kocuria* spp. are rare, they have not been extensively investigated in the past few years.

In this study, a total of three rounds of MADLI-TOF MS were performed to obviate the human error. The results revealed that 11.1 and 27.3% of the bacterial strains isolated from hospital wards and student dormitories could not be identified, respectively, which is a limitation of this study. Given the complexity of environmental microorganisms, mass spectrometric technique and more reference strains, especially for environmental microorganisms, should be developed. The strains identified in this study may indicate the differences between hospital wards and student dormitories, as well as a possibility of imbalance in microbial diversity. Hence, in future research, technologies that are superior to MALDI-TOF MS, such as whole genome sequencing, can be adopted.

## Conclusions

Assessment of environmental quality of hospital wards should be based on comprehensive analysis with multiple indicators. There may be imbalances in the microbial diversity in the hospital wards, therefore, monitoring of the environmental quality of hospitals is important in the prevention of nosocomial infections.

## Additional file

Additional file 1: **Table S3**. Bacterial characterization and proportion of bacterial species identified in hospital wards and student dormitories using MALDI-TOF MS. Description of data: The data have showed the specific proportion of bacterial species identified in hospital wards and student dormitories using MALDI-TOF MS. (DOC 145 kb)

## Data Availability

The datasets used and/or analysed during the current study are available from the corresponding author on reasonable request.

## Abbreviations

ATP: Adenosine triphosphate
CFUs: Colony-forming units
CoNS: Coagulase-negative staphylococci
MALDI-TOF MS: Matrix assisted laser desorption/ionization time-of-flight mass spectrometry
MRSA: Methicillin-resistant Staphylococcus aureus
VRE: Vancomycin-resistant Enterococcus spp

## References

[1] Dancer SJ (2014). Controlling hospital-acquired infection: focus on the role of the environment and new technologies for decontamination. Clin Microbiol Rev.

[2] Weber DJ, Anderson D, Rutala WA (2013). The role of the surface environment in healthcare-associated infections. Curr Opin Infect Dis.

[3] Drees M, Snydman DR, Schmid CH, Barefoot L, Hansjosten K, Vue PM, Cronin M, Nasraway SA, Golan Y (2008). Prior environmental contamination increases the risk of acquisition of vancomycin-resistantenterococci. Clin Infect Dis.

[4] Wilks M, Wilson A, Warwick S, Price E, Kennedy D, Ely A, Millar MR (2006). Control of an outbreak of multidrug-resistant Acinetobacter baumannii-calcoaceticus colonization and infection in an intensive care unit (ICU) without closing the ICU or placing patients in isolation. Infect Control Hosp Epidemiol.

[5] Nseir S, Blazejewski C, Lubret R, Wallet F, Courcol R, Durocher A (2011). Risk of acquiring multidrug-resistant gram-negative bacilli from prior room occupants in the intensive care unit. Clin Microbiol Infect.

[6] Shaughnessy MK, Micielli RL, DePestel DD, Arndt J, Strachan CL, Welch KB, Chenoweth CE (2011). Evaluation of hospital room assignment and acquisition of Clostridium difficile infection. Infect Control Hosp Epidemiol.

[7] Jung C-C, Wu P-C, Tseng CH, Su HJ (2015). Indoor air quality varies with ventilation types and working areas in hospitals. Build Environ.

[8] Kedjarune U, Kukiattrakoon B, Yapong B, Chowanadisai S, Leggat P (2000). Bacterial aerosols in the dental clinic: effect of time, position and type of treatment. Int Dent J.

[9] Mirzaei R, Shahriary E, Qureshi MI, Rakhshkhorshid A, Khammary A, Mohammadi M (2014). Quantitative and qualitative evaluation of bio-aerosols in surgery rooms and emergency department of an educational hospital. Jundishapur J Microbiol.

[10] Okten S, Asan A (2012). Airborne fungi and bacteria in indoor and outdoor environment of the pediatric unit of Edirne government hospital. Environ Monit Assess.

[11] Sautour M, Sixt N, Dalle F, L'Ollivier C, Fourquenet V, Calinon C, Paul K, Valvin S, Maurel A, Aho S, Couillault G, Cachia C, Vagner O, Cuisenier B, Caillot D, Bonnin A (2009). Profiles and seasonal distribution of airborne fungi in indoor and outdoor environments at a French hospital. Sci Total Environ.

[12] Fan J, Li S, Fan C, Bai Z, Yang K (2016). The impact of PM_2.5_ on asthma emergency department visits: a systematic review and meta-analysis. Environ Sci Pollut Res Int.

[13] Han Y, Qi M, Chen Y, Shen H, Liu J, Huang Y, Chen H, Liu W, Wang X, Liu J, Xing B, Tao S (2015). Influences of ambient air PM_2.5_ concentration and meteorological condition on the indoor PM_2.5_ concentrations in a residential apartment in Beijing using a new approach. Environ Pollut.

[14] Pope CA, Dockery DW (2006). Health effects of fine particulate air pollution: lines that connect. J Air Waste Manag Assoc.

[15] Brook RD, Rajagopalan S, Pope CA, Brook JR, Bhatnagar A, Diez-Roux AV, Holguin F, Hong Y, Luepker RV, Mittleman MA, Peters A, Siscovick D, Smith SC, Whitsel L, Kaufman JD (2010). American Heart Association Council on Epidemiology and Prevention, Council on the Kidney in Cardiovascular Disease, and Council on Nutrition, Physical Activity and Metabolism. Particulate matter air pollution and cardiovascular disease: An update to the scientific statement from the American Heart Association. Circulation..

[16] Kioumourtzoglou MA, Schwartz JD, Weisskopf MG, Melly SJ, Wang Y, Dominici F, Zanobetti A (2016). Long-term PM2.5 exposure and neurological hospital admissions in the northeastern United States. Environ Health Perspect.

[17] Erqou S, Clougherty JE, Olafiranye O, Magnani JW, Aiyer A, Tripathy S, Kinnee E, Kip KE, Reis SE (2018). Particulate matter air pollution and racial differences in cardiovascular disease risk. Arterioscler Thromb Vasc Biol.

[18] Puett RC, Hart JE, Yanosky JD, Paciorek C, Schwartz J, Suh H, Speizer FE, Laden F (2009). Chronic fine and coarse particulate exposure, mortality, and coronary heart disease in the Nurses' health study. Environ Health Perspect.

[19] Stafoggia M, Cesaroni G, Peters A, Andersen ZJ, Badaloni C, Beelen R, Caracciolo B, Cyrys J, de Faire U, de Hoogh K (2014). Long-term exposure to ambient air pollution and incidence of cerebrovascular events: results from 11 European cohorts within the ESCAPE project. Environ Health Perspect.

[20] Hamra GB, Guha N, Cohen A, Laden F, Raaschou-Nielsen O, Samet JM, Vineis P, Forastiere F, Saldiva P, Yorifuji T (2014). Outdoor particulate matter exposure and lung cancer: a systematic review and meta-analysis. Environ Health Perspect.

[21] Calderón-Garcidueñas L, Solt AC, Henríquez-Roldán C, Torres-Jardón R, Nuse B, Herritt L, Villarreal-Calderón R, Osnaya N, Stone I, García R (2008). Long-term air pollution exposure is associated with neuroinflammation, an altered innate immune response, disruption of the blood-brain barrier, ultrafine particulate deposition, and accumulation of amyloid beta-42 and alpha-synuclein in children and young adults. Toxicol Pathol.

[22] He K, Yang F, Ma Y, Zhang Q, Yao X, Chan CK, Cadle S, Chan T, Mulawa P (2001). The characteristics of PM2.5 in Beijing, China. Atmos Environ.

[23] Han B, Zhang R, Yang W, Bai Z, Ma Z, Zhang W (2016). Heavy haze episodes in Beijing during January 2013: inorganic ion chemistry and source analysis using highly time-resolved measurements from an urban site. Sci Total Environ.

[24] Wang J, Hang Ho SS, Huang R, Gao M, Liu S, Zhao S, Cao J, Wang G, Shen Z, Han Y (2016). Characterization of parent and oxygenated-polycyclic aromatic hydrocarbons(PAHs) in Xi’an, China during heating period: an investigation of spatial distribution and transformation. Chemosphere..

[25] Loupa G, Zarogianni AM, Karali D, Kosmadakis I, Rapsomanikis S (2016). Indoor/outdoor PM_2.5_ elemental composition and organic fraction medications, in a Greek hospital. Sci Total Environ.

[26] Hart JE, Liao X, Hong B, Puett RC, Yanosky JD, Suh H, Kioumourtzoglou MA, Spiegelman D, Laden F (2015). The association of long-term exposure to PM_2.5_ on all-cause mortality in the Nurses' health study and the impact of measurement-error correction. Environ Health.

[27] Napoli C, Marcotrigiano V, Montagna MT (2012). Air sampling procedures to evaluate microbial contamination: a comparison between active and passive methods in operating theatres. BMC Public Health.

[28] Sanna T, Dallolio L, Raggi A, Mazzetti M, Lorusso G, Zanni A, Farruggia P, Leoni E (2018). ATP bioluminescence assay for evaluating cleaning practices in operating theatres: applicability and limitations. BMC Infect Dis.

[29] Radosevich JL, Wilson WJ, Shinn JH, DeSantis TZ, Andersen GL (2002). Development of a high-volume aerosol collection system for the identification of air-borne micro-organisms. Lett Appl Microbiol.

[30] Toivola M, Alm S, Reponen T, Kolari S, Nevalainen A (2002). Personal exposures and microenvironmental concentrations of particles and bioaerosols. J Environ Monit.

[31] Eduard W, Heederik D (1998). Methods for quantitative assessment of airborne levels of noninfectious microorganisms in highly contaminated work environments. Am Ind Hyg Assoc J.

[32] Croxatto A, Prod'hom G, Greub G (2012). Applications of MALDI-TOF mass spectrometry in clinical diagnostic microbiology. FEMS Microbiol Rev.

[33] Singhal N, Kumar M, Kanaujia PK, Virdi JS (2015). MALDI-TOF mass spectrometry: an emerging technology for microbial identification and diagnosis. Front Microbiol.

[34] Wang F, Meng D, Li X, Tan J (2016). Indoor-outdoor relationships of PM_2.5_ in four residential dwellings in winter in the Yangtze River Delta, China. Environ Pollut.

[35] ASTM Standard E1792–-03(2016). Standard Specification for Wipe Sampling Materials for Lead in Surface Dust. West Conshohocken: ASTM International; 2016. https://www.astm.org.

[36] Yamamoto N, Shendell DG, Peccia J (2011). Assessing allergenic fungi in house dust by floor wipe sampling and quantitative PCR. Indoor Air.

[37] Adams RI, Tian Y, Taylor JW, Bruns TD, Hyvärinen A, Täubel M (2015). Passive dust collectors for assessing airborne microbial material. Microbiome..

[38] Tseng CP, Cheng JC, Tseng CC, Wang C, Chen YL, Chiu DT, Liao HC, Chang SS (2003). Broad-range ribosomal RNA real-time PCR after removal of DNA from reagents: melting profiles for clinically important bacteria. Clin Chem.

[39] Vollmer T, Störmer M, Kleesiek K, Dreier J (2008). Evaluation of novel broad-range real-time PCR assay for rapid detection of human pathogenic fungi in various clinical specimens. J Clin Microbiol.

[40] Cao C, Jiang W, Wang B, Fang J, Lang J, Tian G, Jiang J, Zhu TF (2014). Inhalable microorganisms in Beijing’s PM_2.5_ and PM_10_ pollutants during a severe smog event. Environ Sci Technol.

[41] Liakopoulos V, Petinaki E, Efthimiadi G, Klapsa D, Giannopoulou M, Dovas S, Eleftheriadis T, Mertens PR, Stefanidis I (2008). Clonal relatedness of methicillin-resistant coagulase-negative staphylococci in the haemodialysis unit of a single university Centre in Greece. Nephrol Dial Transplant.

[42] Becker K, Heilmann C, Peters G (2014). Coagulase-negative staphylococci. Clin Microbiol Rev.

[43] Takeuchi F, Watanabe S, Baba T, Yuzawa H, Ito T, Morimoto Y, Kuroda M, Cui L, Takahashi M, Ankai A, Baba S, Fukui S, Lee JC, Hiramatsu K (2005). Whole-genome sequencing of staphylococcus haemolyticus uncovers the extreme plasticity of its genome and the evolution of human-colonizing staphylococcal species. J Bacteriol.

[44] Silva PV, Cruz RS, Keim LS, Paula GR, Carvalho BT, Coelho LR, Carvalho MC, Rosa JM, Figueiredo AM, Teixeira LA (2013). The antimicrobial susceptibility, biofilm formation and genotypic profiles of staphylococcus haemolyticus from bloodstream infections. Mem Inst Oswaldo Cruz.

[45] Barros EM, Ceotto H, Bastos MC, Dos Santos KR, Giambiagi-Demarval M (2012). Staphylococcus haemolyticus as an important hospital pathogen and carrier of methicillin resistance genes. J Clin Microbiol.

[46] Czekaj T, Ciszewski M, Szewczyk EM (2015). Staphylococcus haemolyticus - an emerging threat in the twilight of the antibiotics age. Microbiology..

[47] Froggatt JW, Johnston JL, Galetto DW, Archer GL (1989). Antimicrobial resistance in nosocomial isolates of staphylococcus haemolyticus. Antimicrob Agents Chemother.

[48] Chiew YF, Charles M, Johnstone MC, Thompson KM, Parnell KD, Penno EC (2007). Detection of vancomycin heteroresistant staphylococcus haemolyticus and vancomycin intermediate resistant Staphylococcus epidermidis by means of vancomycin screening agar. Pathology..

[49] Purty S, Saranathan R, Prashanth K, Narayanan K, Asir J, Sheela Devi C, Kumar AS (2013). The expanding spectrum of human infections caused by *Kocuria* species: a case report and literature review. Emerg Microbes Infect.

[50] Savini V, Catavitello C, Masciarelli G, Astolfi D, Balbinot A, Bianco A, Febbo F, D'Amario C, D'Antonio D (2010). Drug sensitivity and clinical impact of members of the genus Kocuria. J Med Microbiol.

[51] Kumar CG, Sujitha P (2014). Kocuran, an exopolysaccharide isolated from Kocuria rosea strain BS-1 and evaluation of its in vitro immunosuppression activities. Enzym Microb Technol.

[52] Kandi V, Palange P, Vaish R, Bhatti AB, Kale V, Kandi MR, Bhoomagiri MR (2016). Emerging bacterial infection: identification and clinical significance of Kocuria species. Cureus..

[53] Chen HM, Chi H, Chiu NC, Huang FY (2015). Kocuria kristinae: a true pathogen in pediatric patients. J Microbiol Immunol Infect.

[54] Moreira JS, Riccetto AG, Silva MT, Vilela MM (2015). Study group Centro Médico de Campinas/Franceschi Medicina laboratorial. Endocarditis by Kocuria rosea in an immunocompetent child. Braz J Infect Dis.

