# Supplementary material for: Evaluation of the complexity of indoor air in hospital wards based on PM2.5, real-time PCR, adenosine triphosphate bioluminescence assay, microbial culture and mass spectrometry
Source: BMC Infect Dis. 2019 Jul 19;19:646. doi: 10.1186/s12879-019-4249-z (PMC6642494; doi:10.1186/s12879-019-4249-z)
Supplement: Supplementary file 1 — Table S3. Bacterial characterization and proportion of bacterial species identified in hospital wards and student dormitories using MALDI-TOF MS. Description of data: The data have showed the specific proportion of bacterial species identified in hospital wards and student dormitories using MALDI-TOF MS. (DOC 145 kb) [file 12879_2019_4249_MOESM1_ESM.doc]

Additional file 1: Table S3 Bacterial characterization and proportion of bacterial species identified in hospital wards and student dormitories using MALDI-TOF MS

| **genera** | **species** | **proportion of bacterial species(%)** | | | | | | | | | |
| --- | --- | --- | --- | --- | --- | --- | --- | --- | --- | --- | --- |
|  |  | General Surgery Dept.Ⅵ | Gastrointestinal Surgery | GI Dept. Ⅰ | ENT Dept. | Nephrology Dept. | Respiration Dept.Ⅰ | Respiration Dept.Ⅲ | Neurology Dept.Ⅵ | Neurology Dept.Ⅶ | Student dormitories |
|  | *S. epidermidis* | 7.5 | 12.5 | 17.5 | 8.7 | 0 | 2.7 | 18.4 | 15.4 | 18.7 | 13.6 |
|  | *S. hominis* | 9.4 | 10.0 | 15.2 | 17.4 | 9.1 | 1.3 | 10.5 | 7.6 | 0 | 36.4 |
|  | *S. capitis* | 0 | 2.5 | 8.7 | 4.3 | 9.1 | 2.7 | 5.3 | 30.8 | 0 | 3.0 |
|  | *S. haemolyticus* | 3.8 | 2.5 | 6.5 | 0 | 9.1 | 22.7 | 2.6 | 0 | 6.3 | 0 |
| *Staphylococcus* | *S .warneri* | 0 | 0 | 0 | 0 | 0 | 1.3 | 7.9 | 0 | 0 | 0 |
|  | *S. caprae* | 0 | 0 | 0 | 0 | 0 | 0 | 0 | 0 | 6.3 | 0 |
|  | *S. pettenkoferi* | 5.7 | 0 | 0 | 0 | 0 | 6.7 | 0 | 0 | 0 | 0 |
|  | *S. simulans* | 0 | 0 | 4.3 | 0 | 0 | 9.3 | 0 | 0 | 0 | 0 |
|  | *S. cohnii* | 0 | 0 | 2.2 | 0 | 0 | 0 | 0 | 0 | 0 | 0 |
| *Micrococcus* | *M. luteus* | 32.1 | 25 | 4.3 | 34.6 | 45.5 | 16.0 | 21.1 | 38.5 | 6.3 | 7.6 |
|  | *M. flavus* | 0 | 2.5 | 0 | 0 | 0 | 0 | 0 | 0 | 0 | 0 |
| *Corynebacterium* | *C. suicordis* | 15.1 | 5.0 | 0 | 8.7 | 0 | 0 | 2.7 | 0 | 6.2 | 0 |
|  | *C.* singulare | 0 | 0 | 0 | 0 | 0 | 0 | 0 | 0 | 6.3 | 0 |
|  | *C. striatum* | 0 | 0 | 0 | 0 | 0 | 0 | 5.3 | 0 | 0 | 0 |
|  | *C. minutissimum* | 11.3 | 0 | 0 | 0 | 0 | 0 | 0 | 0 | 0 | 0 |
|  | *C. mucifaciens* | 1.9 | 0 | 0 | 0 | 0 | 0 | 0 | 0 | 0 | 0 |
|  | *C. aurimucosum* | 0 | 0 | 0 | 0 | 0 | 0 | 0 | 0 | 0 | 0 |
|  | *C. variable* | 0 | 0 | 2.2 | 0 | 0 | 16.0 | 0 | 0 | 0 | 0 |
| *Kocuria* | *K. rhizophila* | 0 | 20.0 | 0 | 0 | 0 | 0 | 0 | 0 | 0 | 0 |
|  | *K. rosea* | 3.8 | 0 | 0 | 0 | 0 | 0 | 0 | 0 | 0 | 0 |
|  | *K. palustris* | 0 | 0 | 0 | 0 | 9.1 | 0 | 2.6 | 0 | 0 | 0 |
|  | *K. marina* | 1.9 | 0 | 0 | 0 | 9.1 | 0 | 0 | 0 | 0 | 0 |
| *Bacillus* | *B. circulans* | 0 | 0 | 0 | 4.3 | 0 | 0 | 0 | 0 | 0 | 0 |
|  | *B. megaterium* | 0 | 0 | 6.5 | 0 | 0 | 0 | 0 | 0 | 0 | 4.5 |
|  | *B. simplex* | 0 | 2.5 | 0 | 4.3 | 0 | 0 | 0 | 0 | 0 | 0 |
|  | *B. niacini* | 0 | 0 | 0 | 0 | 0 | 0 | 2.6 | 0 | 0 | 0 |
|  | *B. altitudinis* | 0 | 0 | 0 | 0 | 0 | 0 | 0 | 0 | 6.3 | 0 |
|  | *B. silvestris* | 0 | 0 | 0 | 0 | 0 | 0 | 0 | 0 | 6.2 | 0 |
| *Streptococcus* | *S. oralis* | 0 | 2.5 | 2.2 | 0 | 0 | 4.0 | 0 | 0 | 0 | 0 |
| *Moraxella* | *M. osloensis* | 1.9 | 0 | 2.2 | 9 | 0 | 0 | 0 | 0 | 6.3 | 7.6 |
| *Enterococcus* | *E. faecium* | 0 | 0 | 0 | 0 | 0 | 0 | 7.9 | 0 | 0 | 0 |
|  | *E. faecalis* | 0 | 2.5 | 0 | 0 | 0 | 0 | 0 | 0 | 0 | 0 |
| *Acinetobacter* | *A. radioresistens* | 0 | 0 | 0 | 0 | 0 | 0 | 0 | 0 | 18.8 | 0 |
|  | *A. johnsonii* | 0 | 0 | 0 | 0 | 0 | 1.3 | 0 | 0 | 0 | 0 |
| *Aerococcus* | *A. viridans* | 0 | 0 | 0 | 8.7 | 0 | 1.3 | 0 | 0 | 0 | 0 |
| Other bacillus | *C. cellulans* | 0 | 0 | 4.3 | 0 | 0 | 0 | 0 | 0 | 0 | 0 |
|  | *P. oryzihabitans* | 0 | 2.5 | 0 | 0 | 0 | 0 | 0 | 0 | 0 | 0 |
|  | *E. aurantiacum* | 0 | 0 | 0 | 0 | 0 | 0 | 2.6 | 0 | 0 | 0 |
|  | *C. flaccumfaciens* | 0 | 2.5 | 0 | 0 | 0 | 0 | 0 | 0 | 0 | 0 |
|  | *A. pascens* | 0 | 0 | 0 | 0 | 0 | 0 | 0 | 0 | 6 | 0 |
| Unidentified | Unidentified | 5.6 | 7.5 | 23.9 | 0 | 9.0 | 14.7 | 10.5 | 7.7 | 6.3 | 27.3 |
| Total (%) |  | 100 | 100 | 100 | 100 | 100 | 100 | 100 | 100 | 100 | 100 |
